# Supplementary material for: Rieske FeS overexpression in tobacco provides increased abundance and activity of cytochrome b 6 f
Source: Physiol Plant. 2022 Nov 4;174(6):e13803. doi: 10.1111/ppl.13803 (PMC9828649; doi:10.1111/ppl.13803)
Supplement: Supplementary file 1 — Figure S1. Schematic representation of the gene construct used for Rieske overexpression. LB, T‐DNA left border; 35 S, cauliflower mosaic virus 35 S promoter; HPT, hygromycin phosphotransferase gene; pRbcS2B, A. thaliana Rubisco small subunit 2B (AT5G38420) promoter; AtPetC, A. thaliana PetC (AT4G03280) coding sequence; thsp, A. thaliana HEAT SHOCK PROTEIN 18.2 ( AT5G59720 ) terminator; pNOS, nopaline synthase promoter; bar, bialaphos resistance gene; RB, T‐DNA right border. Figure S2. Blue Native‐PAGE and blots performed in this study. Relative quantification of western blot signals is shown in Figure 2. Figure S3. Linear fits of the dilution series from western blots and ponceau stained membranes (Figure 4). The transgenic samples (100% loading) are displayed as Mean ± SE (n = 3). Figure S4. Comparison of the thylakoid CytF content in plants grown in a commercial soil mix supplemented with 2 g L−1 osmocote (mix 1) and a homemade soil mix supplemented with 7 g L−1 osmocote (mix 2). Mean ± SE, n = 3 biological replicates for mix 1, n = 5 for mix 2. Not significant (t test, p = 0.63). Figure S5. Fluorescent transients obtained by LIFT analysis from WT (black traces) and Rieske‐OE plants of lines R17, R25 and R26. Two LIFT‐FRR duty cycles are presented for each line. FR, the last point at the end of far‐red illumination, prior to switching on the actinic light; FR + AL, the point measured after 3 min of illumination with far‐red and actinic light. Figure S6. Schematic representation of experimental design for the field experiments. A randomised complete block design was used for both experiments where each block had 4 plants for every line. Rows were randomised using RAND function (Microsoft Excel 2010). Both experiments were surrounded by a WT border and each separate experiment was bordered by two lines of WT. Figure S7. CO2 response of (a) CO2 assimilation, A, and (b) the effective quantum yield of PSII, Y(II), of Illinois field‐grown control and Rieske‐OE plants m [file PPL-174-0-s001.pdf]

Supplementary materials

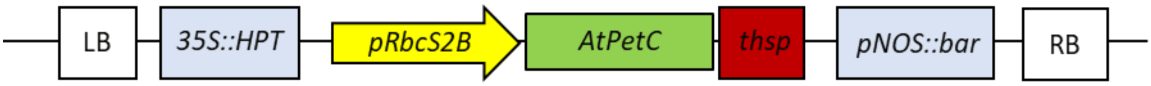

Fig. S1. Schematic representation of the gene construct used for Rieske overexpression. LB, T-DNA left border; *35S*, cauliflower mosaic virus *35S* promoter; *HPT*, hygromycin phosphotransferase gene; *pRbcS2B*, *A. thaliana* Rubisco small subunit 2B (*AT5G38420*) promoter; *AtPetC*, *A. thaliana PetC* (AT4G03280) coding sequence; *thsp*, *A. thaliana HEAT SHOCK PROTEIN 18.2* (*AT5G59720*) terminator; *pNOS*, nopaline synthase promoter; *bar*, bialaphos resistance gene; RB, T-DNA right border.

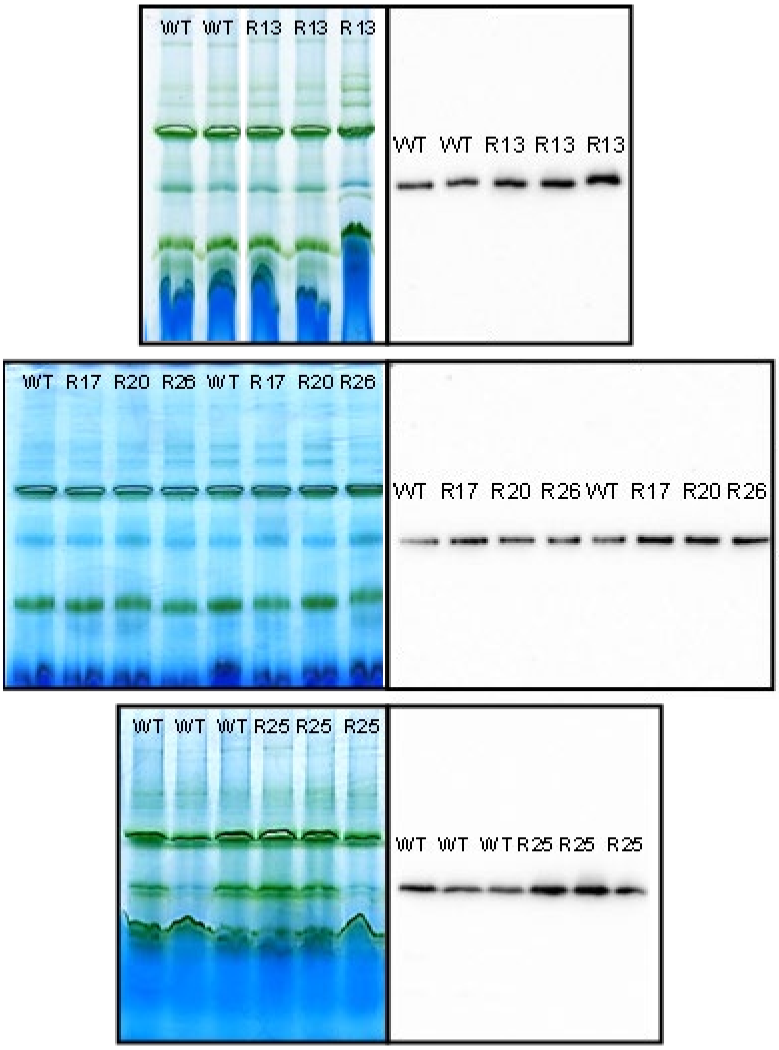

Fig. S2. BN-PAGE and blots performed in this study. Relative quantification of western blot signals is shown in Fig 2.

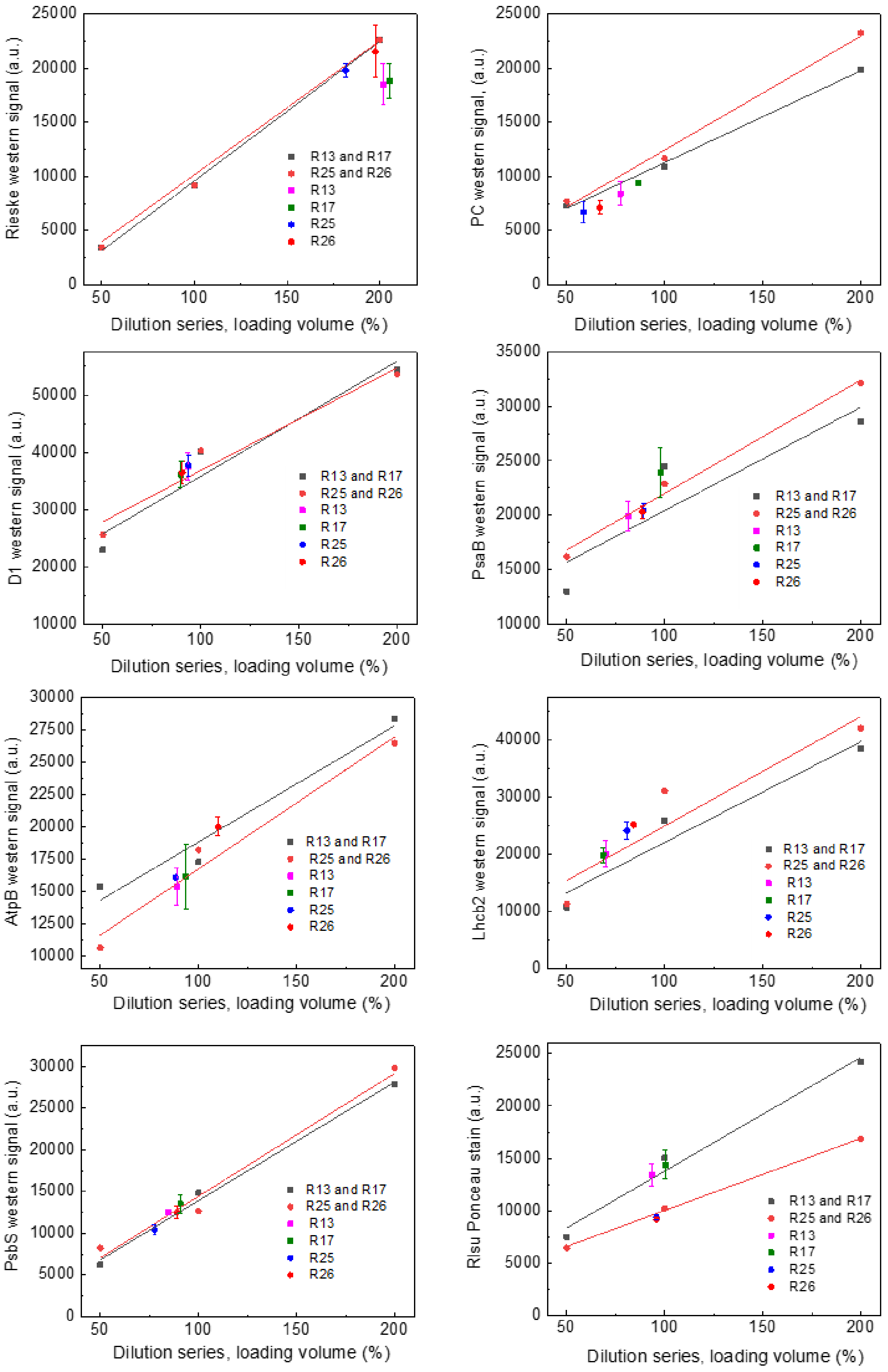

Fig S3. Linear fits of the dilution series of leaf disc western blots and ponceau stain (Fig.4). The transgenic samples (100% loading) are displayed with SE (n+3).

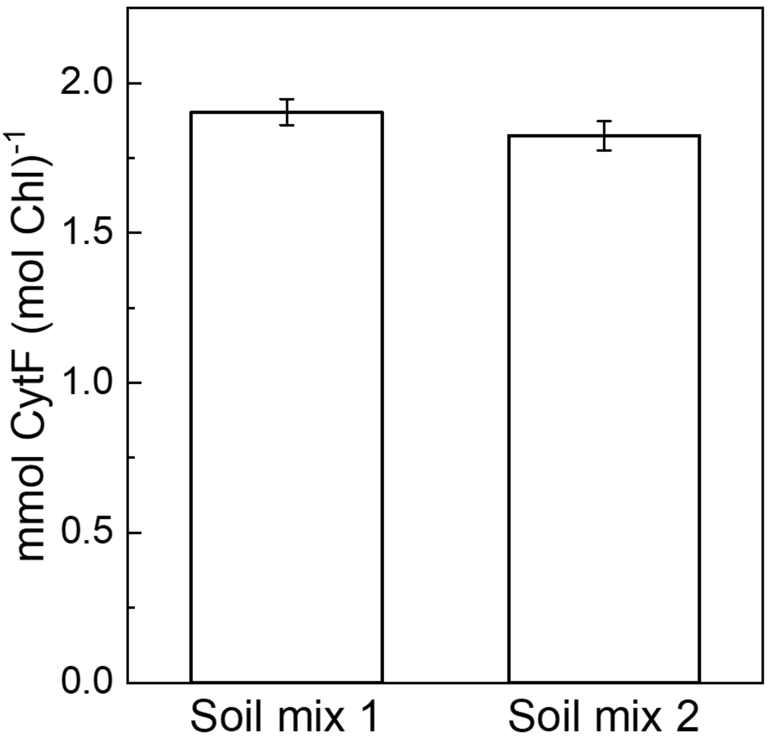

Fig S4. Comparison of the thylakoid CytF content in plants grown in a commercial soil mix supplemented with 2 g L^{-1} osmocote (mix 1) and a homemade soil mix supplemented with 7 g L^{-1} osmocote (mix 2). Mean ± SE, n = 3 biological replicates for mix 1, n = 5 for mix 2. Not significant (t-test, P = 0.63).

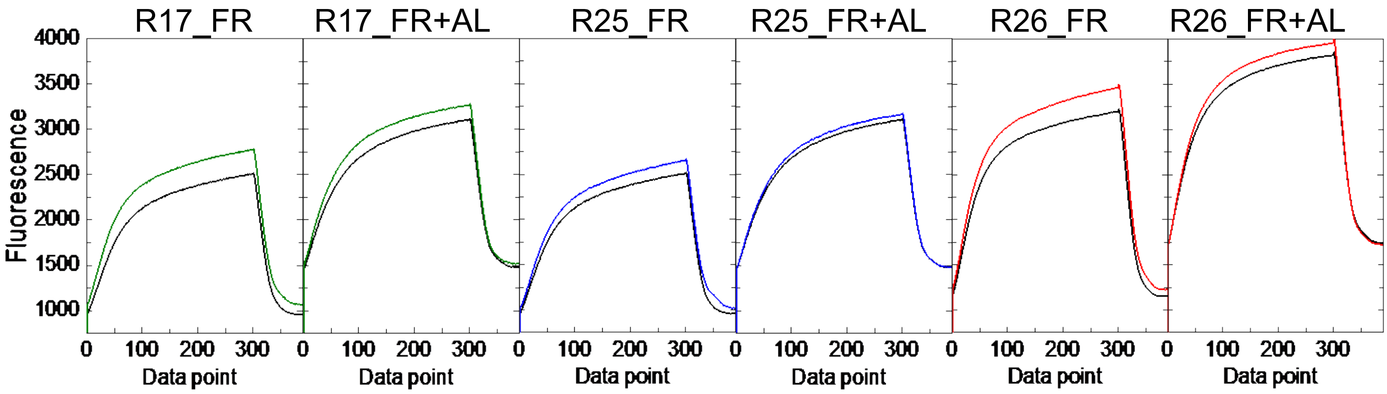

Fig. S5. Fluorescent transients obtained by LIFT analysis from WT (black traces) and Rieske-OE plants of lines R17, R25 and R26. Two LIFT-FRR duty cycles are presented for each line. FR, the last point at the end of far-red illumination, prior to switching on the actinic light; FR+AL, the point measured after 3 minutes of illumination with far-red and actinic light.

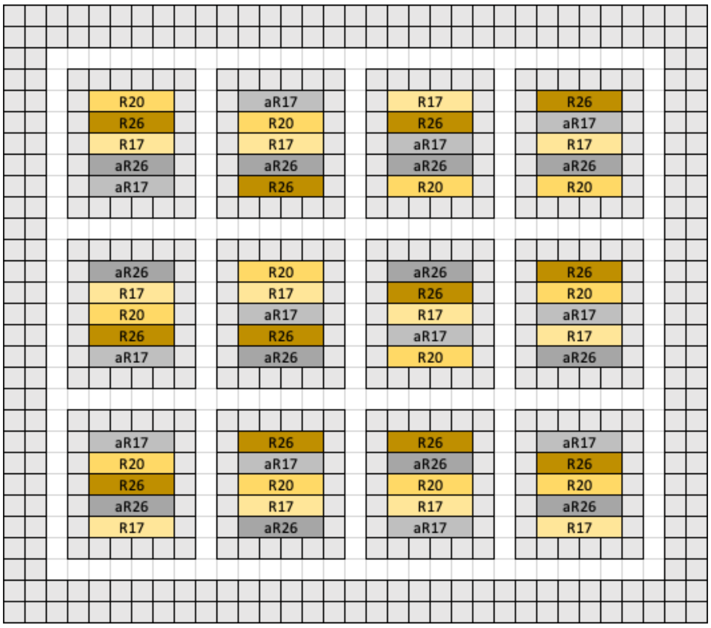

Fig. S6. Schematic representation of experimental design for the field experiments. A randomized complete block design was used for both experiments where each block had 4 plants for every line. Rows were randomised using RAND function (Microsoft Excel 2010). Both experiments were surrounded by a WT border and each separate experiment was bordered by two lines of WT.

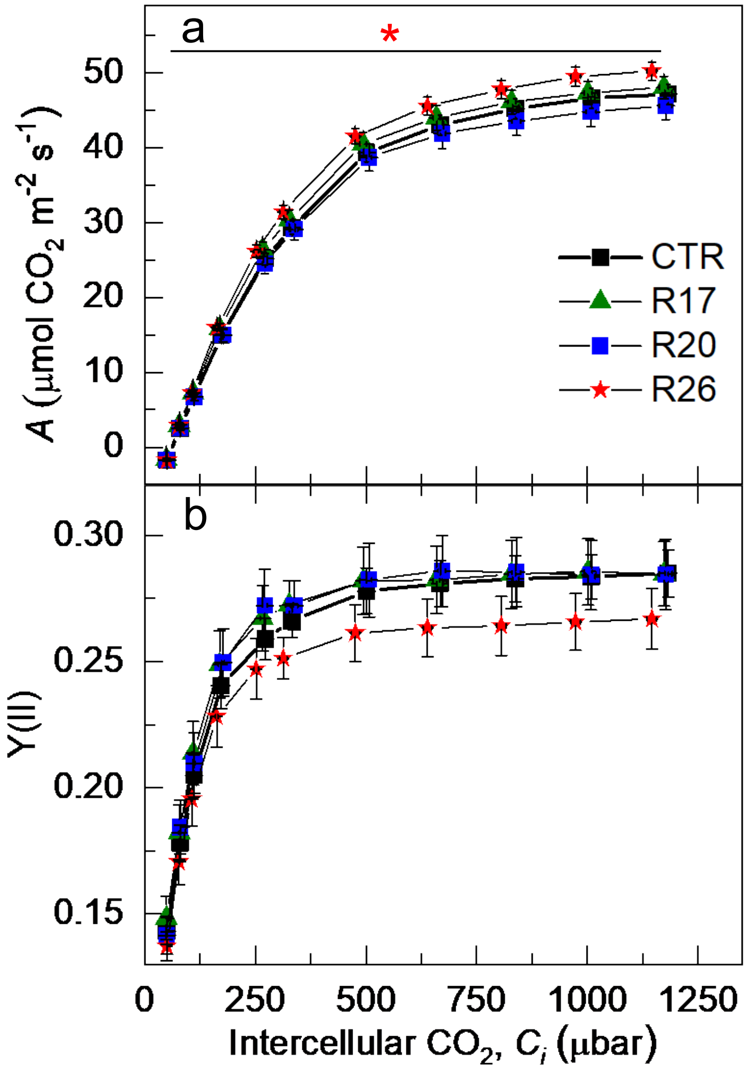

Fig. S7. CO_2 response of (**a**) CO_2 assimilation, *A*, and (**b**) the effective quantum yield of PSII, Y(II), of Illinois field-grown control and Rieske-OE plants measured at 2000 μmol m^{-2} s^{-1}. The control group (CTR) represents both WT and azygous plants. Mean ± SE, $n = 18$ for CTR plants, $n = 12$ for transgenic lines. No consistently significant differences were found between the genotypes and control plants (linear mixed-effects model and type II ANOVA, $P < 0.05$).
